# Letermovir Prophylaxis for Cytomegalovirus Infection in Adult Allogeneic Stem Cell Transplantation: A Retrospective Analysis From a Lower-Middle-Income Country

**DOI:** 10.7759/cureus.102136

**Published:** 2026-01-23

**Authors:** Neha Rastogi, Swati Bhayana, Kapil Chahal, Nikhil M Kumar, Anusha Swaminathan, Ashutosh Panda, Suyash Bharat, Onyeaghala Chizaram, Shrinidhi Nathany, Anindita Paul, Rachit Agrawal, Vikas Dua, Rahul Bhargava

**Affiliations:** 1 Infectious Diseases, Fortis Memorial Research Institute, Gurugram, IND; 2 Haematology, Fortis Memorial Research Institute, Gurugram, IND; 3 Haematology, Fortis Hospital, Gurugram, Gurgaon, IND; 4 Infectious Diseases, International Centre for Genetic Engineering and Biotechnology, New Delhi, IND; 5 Medical Affairs, Zydus Lifesciences Limited, Ahmedabad, IND; 6 Internal Medicine/Infectious Diseases, University of Port Harcourt Teaching Hospital, Port Harcourt, NGA; 7 Paediatric Hematology, Fortis Memorial Research Institute, Gurugram, IND

**Keywords:** bioequivalent, cmv prophylaxis, cytomegalovirus, hematopoietic stem cell transplantation, india, letermovir, real-world study

## Abstract

Background: Cytomegalovirus (CMV) reactivation remains a leading cause of morbidity and non-relapse mortality following allogeneic hematopoietic stem cell transplantation (HSCT), especially in high-seroprevalence, resource-limited settings. Letermovir has significantly improved CMV prophylaxis by providing effective antiviral protection without hematologic or renal toxicity. The present study provides the first real-world comparative data of innovator and bioequivalent letermovir formulations for CMV prophylaxis in adult allogenic HSCT recipients from India.

Methods: This retrospective study was conducted at the Adult Hematology and Bone Marrow Transplant Unit, Fortis Memorial Research Institute, Gurugram, India. It included 29 CMV-seropositive patients’ data of allogeneic HSCT recipients who received letermovir prophylaxis from day +7 to day +100 (innovator n = 7; bioequivalent n = 22). Treatment allocation was based on institutional availability and consultant discretion, with baseline characteristics comparable in both groups. Weekly quantitative CMV PCR monitoring was performed during prophylaxis, with continued surveillance thereafter as per institutional practice. The primary endpoint was CMV-free survival through day +100. Secondary endpoints included CMV reactivation incidence, time to CMV breakthrough, and overall survival. Statistical analyses were performed using IBM SPSS Statistics for Windows version 25.0, with *p* < 0.05 considered significant.

Results: The mean recipient age in the analysed data was 34.1 ± 13.2 years, with 82.8% males; 62% patients that underwent haploidentical transplants. CMV reactivation data showed occurrence in six patients (20.7%) overall, four (13.8%) in the bioequivalent group, and two (28.6%) in the innovator group (*p* = 0.56). Two patients in the bioequivalent group developed CMV reactivation after completion of prophylaxis at days 121 and 130, respectively. The overall median duration to CMV breakthrough was 121 days as observed (121 vs. 84 days; log-rank = 1.88; *p* = 0.17).

Conclusion: No statistically significant differences were observed between innovator and bioequivalent letermovir formulations with respect to safety or CMV outcomes. Availability of a cost-effective bioequivalent formulation may substantially expand access to CMV prophylaxis, supporting improved transplant outcomes in high-burden LMICs.

## Introduction

Cytomegalovirus (CMV) reactivation remains a major cause of morbidity and non-relapse mortality (NRM) following allogeneic hematopoietic stem cell transplantation (HSCT). Despite advances in transplant care, 40-70% of CMV-seropositive recipients develop viral reactivation within the first 100 days post-transplant, often leading to graft failure, severe pneumonitis, gastrointestinal disease, and multi-organ dysfunction that significantly impact survival outcomes [1,2]. The risk is further amplified in recipients of haploidentical or HLA-mismatched grafts, those with grade ≥2 graft-versus-host disease (GVHD), or under prolonged corticosteroid therapy [3,4].

Conventional CMV management strategies rely predominantly on pre-emptive therapy with antivirals such as ganciclovir, valganciclovir, and foscarnet [5,6]; however, their use is frequently limited by myelosuppression, nephrotoxicity, and the logistical challenges of intensive monitoring, particularly during the early post-transplant phase [7]. Other prophylactic agents, including acyclovir, valacyclovir, maribavir, brincidofovir, and intravenous immunoglobulin, have demonstrated variable and often limited efficacy [8,9]. These limitations are more pronounced in lower-middle-income countries (LMICs), where CMV seroprevalence is high, and supportive care resources to manage drug-related toxicities remain scarce.

Letermovir, a novel CMV DNA terminase complex inhibitor, acts by targeting the viral UL56 subunit, thereby preventing DNA cleavage and packaging without affecting host polymerases [10,11]. Since its approval by the U.S. FDA and EMA in 2017 for CMV prophylaxis in seropositive allogeneic HSCT recipients, letermovir has consistently demonstrated efficacy in reducing CMV reactivation and CMV-related mortality, with an excellent hematologic and renal safety profile [12,13]. Subsequent real-world and systematic reviews encompassing over 40 studies have reaffirmed its benefit, with pooled odds ratios for CMV reactivation ranging from 0.13 to 0.24 (P < 0.05).

However, data from LMIC settings remain limited, where both CMV burden and treatment access constraints are unique. The recent introduction of a cost-effective, bioequivalent generic formulation of letermovir (ANVIMO®) offers a promising alternative to the innovator drug, potentially improving accessibility without compromising safety or efficacy. The present study provides the first comparative evaluation data from an Indian cohort examining the feasibility and clinical performance of innovator versus bioequivalent letermovir prophylaxis in adult allogeneic HSCT recipients. The primary focus is on a CMV-free post-transplant period and breakthrough rates, with secondary assessments of overall survival, breakthrough timing, and tolerability within the early post-transplant period.

## Materials and methods

Study design and setting

This retrospective study was conducted at the Adult Hematology and Bone Marrow Transplant Unit, Fortis Memorial Research Institute, Gurugram, India. We reviewed medical records of adult recipients of allogeneic HSCT who received letermovir prophylaxis for prevention of CMV reactivation as part of routine clinical care. Both the innovator formulation (Prevymis®, Merck) and a bioequivalent generic formulation (ANVIMO®, Zydus) approved by the Central Drugs Standard Control Organization (CDSCO) were evaluated using existing institutional records. Selection of innovator versus bioequivalent formulation was driven by institutional procurement availability and operational practices rather than patient-specific CMV risk stratification. Given the small sample size and limited number of outcome events, formal multivariable adjustment for potential confounders was not performed; this limitation is acknowledged. Patients were identified consecutively from medical records, and outcomes were ascertained through review of standardized post-transplant CMV surveillance and follow-up documentation. Bioequivalence of the generic formulation was based on regulatory approval supported by published pharmacokinetic data, rather than independent pharmacokinetic testing within the present study.

Study population

Data from 29 adult patients were retrieved from medical records and analyzed, including completed data of patients who visited the facility over a period of one year (September 2024-2025). Seven patients received the innovator brand and 22 received the bioequivalent letermovir formulation. All patients were at high risk for CMV reactivation because of their underlying hematologic malignancies, donor type, or intensity of immunosuppressive therapy. Data of CMV-seropositive adults aged 18 years or above who underwent allogeneic HSCT and initiated letermovir prophylaxis from the seventh day post transplantation, as per previous studies, were included. Patients’ data with donor-recipient CMV sero-negativity, pre-existing CMV infection before initiation of prophylaxis, or those who died or discontinued therapy before starting letermovir were excluded.

Intervention protocol

Letermovir prophylaxis was initiated uniformly on day +7 post-HSCT and continued for up to 100 days, in accordance with the U.S. FDA-approved dosing regimen (480 mg once daily orally, or 240 mg once daily when co-administered with cyclosporine). Dose modification or discontinuation was permitted at the treating physician’s discretion in the event of CMV breakthrough infection, intolerance, or clinically significant toxicity. Concomitant immunosuppressive agents such as tacrolimus or cyclosporine were given according to institutional protocol. Use of other antivirals, such as acyclovir or valacyclovir, was based on physician discretion and supportive-care policy. Conditioning regimens were tailored to the underlying disease and donor profile, with individualized decisions made by the treating haematologist.

Study endpoints

The primary endpoint was CMV-free survival through day +100 post-transplantation. Secondary endpoints included incidence of CMV reactivation during and after prophylaxis, time to CMV breakthrough, and all-cause mortality during follow-up.

CMV surveillance and definitions

CMV monitoring was performed using quantitative polymerase chain reaction (PCR) assays on whole blood samples, reported in copies/mL. Surveillance was conducted weekly from day +7 until completion of prophylaxis at day +100, with continued monitoring thereafter based on institutional protocol and clinical discretion. CMV reactivation was defined as detectable CMV DNAemia above the assay's lower limit of detection. Decisions regarding initiation of pre-emptive therapy were made by the treating physician based on viral load kinetics, clinical status, and degree of immunosuppression.

Statistical analysis

All analyses were performed using IBM SPSS Statistics v25. Continuous variables were summarized as mean ± SD or median (IQR), and categorical variables as frequencies (%). Group comparisons used Chi-square/Fisher’s exact tests for categorical and t-test/Mann-Whitney U for continuous data. Survival analyses for overall and CMV breakthrough survival were conducted using Kaplan-Meier curves with Log-Rank testing. A p < 0.05 was considered statistically significant.

Ethical considerations

This study did not require separate institutional ethics committee approval, as letermovir was prescribed in accordance with established guidelines and as part of the standard of care for allogeneic HSCT recipients.

## Results

A total of 29 CMV-seropositive adult allogeneic HSCT recipients were identified from available records, and their data were analyzed. Of this, 22 (75.9%) received the bioequivalent formulation, and seven (24.1%) received the innovator formulation of letermovir. The mean age of the patients was 34.14 ± 13.2 years, and 82.8% (n = 24) were males. Acute myeloid leukemia (AML) was the most common indication for transplantation, accounting for 13 patients overall (six in the innovator group and seven in the bioequivalent group). Other indications included myelodysplastic syndromes (MDS; n = 4), chronic myeloid leukemia in blast crisis (n = 2), and acute lymphoblastic leukemia (n = 7), comprising Philadelphia chromosome-positive ALL (n = 1), B-cell ALL (n = 4), and ETP-ALL (n = 2). Additional diagnoses included B-cell lymphoma (n = 2) and severe aplastic anemia (n = 1). Baseline demographic and transplant characteristics were comparable between the innovator and bioequivalent letermovir groups (Table 1).

**Table 1:** Baseline demographic and donor characteristics of the study population (N = 29)

Variable	Category	Frequency (n)	Percentage (%)
Recipient gender	Male	24	82.76
	Female	5	17.24
Donor gender	Male	19	65.52
	Female	10	34.48
Type of donor	Haploidentical	18	62.1
	HLA-matched sibling	10	34.5
	Matched unrelated	1	3.4
Mean recipient age (years)		34.14 ± 13.2	—

As per the available transplant records, patients received diagnosis-adapted but protocolized conditioning regimens according to institutional practice. Myeloid malignancies (AML and MDS) predominantly received fludarabine-based conditioning, most commonly fludarabine-treosulfan (FLU/Treo) with or without antithymocyte globulin (ATG) or fludarabine-melphalan (FLU/Mel), with low-dose total body irradiation (2-4 Gy) incorporated in selected high-risk cases. Lymphoid malignancies, including ALL and lymphomas, more frequently received fludarabine-based regimens incorporating cyclophosphamide, thiotepa, melphalan, and/or higher-dose TBI (up to 8 Gy). Patients with CML and Ph+ ALL had received fludarabine-based conditioning following standard disease-directed therapy, including prior tyrosine kinase inhibitor exposure where applicable. GVHD prophylaxis was tailored to donor type and conditioning intensity and included post-transplant cyclophosphamide-based regimens combined with cyclosporine and mycophenolate mofetil, or calcineurin inhibitor-based prophylaxis with methotrexate. The distribution of conditioning strategies and GVHD prophylaxis was comparable between the innovator and bioequivalent letermovir groups, ensuring similar degrees of transplant-related immunosuppression.

Neutrophil engraftment was documented in the records of all patients, typically occurring between day +14 and day +18 post-transplant; no episodes of graft failure were identified. Baseline demographic and transplant characteristics were comparable between the innovator and bioequivalent groups. Data showed that Letermovir prophylaxis was initiated uniformly on day +7 post-transplantation for all study participants and continued for a median duration of 93 days (range = 90-100 days). Both innovator and bioequivalent formulations were administered according to standard dosing recommendations, and there were no instances of treatment discontinuation attributable to intolerance or toxicity throughout the prophylaxis period. Review of the available records demonstrated excellent overall adherence, with all patients completing the intended course of therapy or discontinuing only at physician-defined clinical endpoints (Table 2).

**Table 2:** Letermovir prophylaxis characteristics among study participants (N = 29)

Parameter	Innovator (n = 7)	Bioequivalent (n = 22)	Total (N = 29)
Initiation day	Day +7 in all	Day +7 in all	Day +7 in all
Median duration of prophylaxis (days)	100 (93–100)	93 (90–100)	93 (90–100)
Discontinuation due to intolerance/toxicity	0 (0%)	0 (0%)	0 (0%)
Completion of prophylaxis	7 (100%)	22 (100%)	29 (100%)

CMV reactivation was identified overall in 6/29 patients (20.7%), with 4/22 (13.79%) in the bioequivalent group and 2/7 (28.57%) in the innovator group. The median time to CMV breakthrough for the study population was 121 days, with time-to-breakthrough distributions showing 121 days in the bioequivalent group versus 84 days in the innovator group (Viral load ranged from 11,000 to 12,000 copies/ml). Two patients in the bioequivalent cohort developed CMV reactivation after completion of prophylaxis, occurring at days 121 and 130, respectively, highlighting the risk of delayed CMV rebound post-therapy. Importantly, three out of six CMV reactivation events occurred in patients with acute GVHD receiving intensified immunosuppression, underscoring immune suppression as the primary driver of CMV risk rather than drug formulation effectiveness (Table 3).

**Table 3:** Comparison of cytomegalovirus (CMV) reactivation between innovator and bioequivalent letermovir groups (N = 29)

CMV reactivation	Bioequivalent (n = 22)	Innovator (n = 7)	Test Statistic	p-value
Yes	4 (13.79%)	2 (28.57%)	χ² = 0.337	0.5614
No	18 (86.21%)	5 (71.43%)		
Total	22	7		

Across the combined cohort, acute GVHD occurred in 5/29 patients (17.2%), all following haploidentical HSCT. Of these, four developed grade 2 lower gastrointestinal GVHD, and three experienced severe grade 3-4 GI GVHD requiring multi-agent therapy including corticosteroids, ruxolitinib, etanercept, and vedolizumab. None of the patients in the innovator group developed GVHD-associated complications such as BK virus reactivation, whereas the bioequivalent cohort recorded BK virus infection in two patients (9.09%) (Table 4).

**Table 4:** Post-transplant complications among study participants (N = 29)

Complication	Bioequivalent (n = 22)	Innovator (n = 7)	Overall (N = 29)
Graft-versus-host disease (GVHD)	5 (22.73%)	0 (0%)	5 (17.24%)
BK-virus infection	2 (9.09%)	0 (0%)	2 (6.9%)
Letermovir-related discontinuation	0 (0%)	0 (0%)	0 (0%)

**Figure 1:**
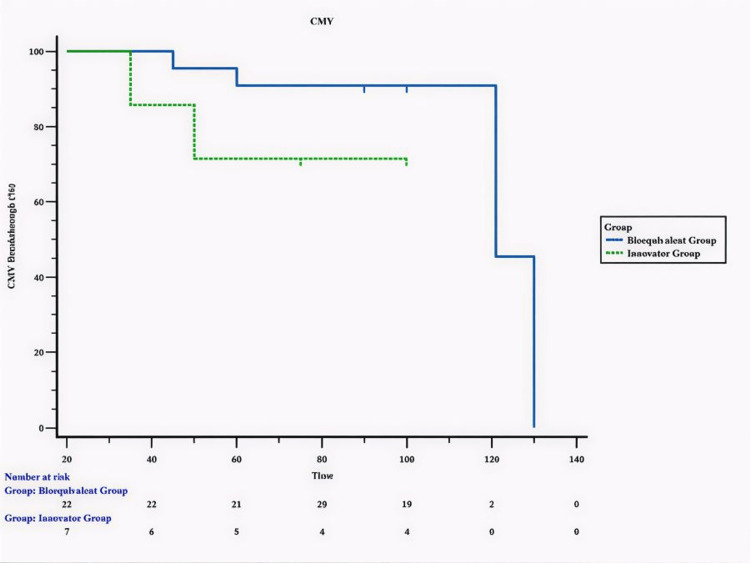
Kaplan–Meier survival curve comparing time to cytomegalovirus (CMV) breakthrough between the innovator and bioequivalent letermovir groups in adult allogeneic hematopoietic stem cell transplantation (HSCT) recipients

Kaplan-Meier survival analysis demonstrated no statistically significant difference in CMV breakthrough timing between the two groups (log-rank test = 1.88; p = 0.17), confirming comparable efficacy of the innovator and bioequivalent formulations. Overall reported survival was high, with 27/29 patients (93.1%) alive at last follow-up. Two deaths (6.9%) occurred, one in each group, both in the setting of grade 4 GI GVHD with CMV breakthrough despite combination immunosuppression (Figure 1).

Taken together, these findings indicate that no statistically significant differences were observed in CMV reactivation rates or breakthrough timing between innovator and bioequivalent letermovir formulations in this small cohort. CMV risk appeared to be primarily associated with the severity of immunosuppression and acute GVHD rather than formulation type.

## Discussion

CMV reactivation remains one of the leading infectious complications after allogeneic HSCT, contributing significantly to non-relapse mortality and post-transplant morbidity despite improvements in supportive care [4]. This retrospective study represents the first comparative experience from India evaluating both the innovator and bioequivalent letermovir formulations for CMV prophylaxis in high-risk adult HSCT recipients. Data showed that Letermovir prophylaxis was uniformly initiated on day +7 and continued for a median of 93 days. No treatment discontinuations or drug-related toxicities occurred in either arm, underscoring the favourable tolerability of this antiviral. CMV reactivation occurred in 20.7% overall, 13.8% in the bioequivalent group, and 28.6% in the innovator group (p = 0.56). Median survival was identical at 100 days in both groups (log rank = 1.2638; p = 0.26). CMV breakthrough occurred later in the bioequivalent group (median 121 days) than in the innovator group (84 days), although this difference was not statistically significant (log rank = 1.88; p = 0.17). Notably, all breakthrough infections occurred after completion of prophylaxis, consistent with late CMV reactivation patterns reported in prior studies [14,15].

The efficacy and safety of letermovir as primary CMV prophylaxis have been well established since the pivotal randomized trial by Marty et al. [3], which demonstrated a significant reduction in clinically significant CMV infection (50.0-19.1%; p < 0.001). Subsequent real-world and meta-analytic data [1,13] corroborate these findings, confirming low reactivation rates and excellent safety profiles. Data from our study extends these observations to an LMIC setting characterized by a predominance of haploidentical donors (62%) and extensive immunosuppression, showing that both formulations offer equivalent protection without hematologic or renal toxicity consistent with letermovir’s selective mechanism targeting the viral UL56 terminase complex [10,12]. All CMV breakthroughs identified in this analysis occurred after discontinuation of prophylaxis, highlighting the ongoing risk of late CMV reactivation. These results reinforce previous findings by Liu et al. (2022) and Lin et al. (2023) [14,15], who observed increased CMV events after letermovir withdrawal at day +100, particularly in patients with delayed immune reconstitution or those receiving post-transplant cyclophosphamide. Our findings suggest that extended or individualized prophylaxis, guided by immune recovery monitoring, may be beneficial in such high-risk populations.

The introduction of a bioequivalent generic letermovir approved by the CDSCO in 2024 [11] carries important clinical and economic implications. Demonstrated pharmacokinetic equivalence and the comparable clinical outcomes observed here support its use as an effective and affordable alternative to the innovator product. Given India’s high CMV seroprevalence (>85%) and increasing reliance on haploidentical transplants [16], widespread access to this formulation could significantly reduce reliance on myelotoxic antivirals and CMV-related hospitalizations, aligning with international guidelines [5].

Together, these findings from medical record review suggest that both innovator and bioequivalent letermovir were safe and well tolerated, with no observed clinically meaningful differences in CMV-free survival or timing of CMV breakthrough during the early post-HSCT period. The occurrence of delayed CMV reactivation after prophylaxis completion highlights the need for continued CMV surveillance beyond day +100 and supports further evaluation of extended-duration or risk-adapted prophylaxis strategies in future multicentre studies from low- and middle-income countries (LMICs). This study was conducted at a single center and included a small cohort with unequal group sizes, limiting statistical power to detect modest differences in efficacy outcomes. Although prophylaxis initiation and monitoring protocols were uniform, the study was not powered to establish equivalence or non-inferiority between formulations. Accordingly, the absence of statistically significant differences should be interpreted as an observational finding rather than evidence of therapeutic equivalence. Residual confounding related to donor type, conditioning intensity, GVHD prophylaxis, and post-transplant immunosuppression cannot be excluded and should be considered when interpreting these results.

## Conclusions

In this study, no statistically significant differences were observed between innovator and bioequivalent letermovir formulations with respect to CMV reactivation, breakthrough timing, or safety in adult allogeneic HSCT recipients. These findings support the feasibility of using a cost-effective bioequivalent formulation in routine practice in resource-limited settings. However, larger prospective studies with adequate power and adjustment for key CMV risk factors are required to confirm comparative effectiveness.
